# Does Assessment Type Matter? A Measurement Invariance Analysis of Online and Paper and Pencil Assessment of the Community Assessment of Psychic Experiences (CAPE)

**DOI:** 10.1371/journal.pone.0084011

**Published:** 2014-01-22

**Authors:** Marloes Vleeschouwer, Chris D. Schubart, Cecile Henquet, Inez Myin-Germeys, Willemijn A. van Gastel, Manon H. J. Hillegers, Jim J. van Os, Marco P. M. Boks, Eske M. Derks

**Affiliations:** 1 Brain Center Rudolf Magnus, Department of Psychiatry, University Medical Center Utrecht, Utrecht, The Netherlands; 2 Department of Psychiatry, Academic Medical Center Amsterdam, Amsterdam, The Netherlands; 3 Department of Psychiatry and Psychology, School of Mental Health and Neuroscience, Maastricht University Medical Centre, Maastricht, The Netherlands; Research and Development Corporation, United States of America

## Abstract

**Background:**

The psychometric properties of an online test are not necessarily identical to its paper and pencil original. The aim of this study is to test whether the factor structure of the Community Assessment of Psychic Experiences (CAPE) is measurement invariant with respect to online vs. paper and pencil assessment.

**Method:**

The factor structure of CAPE items assessed by paper and pencil (N = 796) was compared with the factor structure of CAPE items assessed by the Internet (N = 21,590) using formal tests for Measurement Invariance (MI). The effect size was calculated by estimating the Signed Item Difference in the Sample (SIDS) index and the Signed Test Difference in the Sample (STDS) for a hypothetical subject who scores 2 standard deviations above average on the latent dimensions.

**Results:**

The more restricted Metric Invariance model showed a significantly worse fit compared to the less restricted Configural Invariance model (χ^2^(23) = 152.75, p<0.001). However, the SIDS indices appear to be small, with an average of −0.11. A STDS of −4.80 indicates that Internet sample members who score 2 standard deviations above average would be expected to score 4.80 points lower on the CAPE total scale (ranging from 42 to 114 points) than would members of the Paper sample with the same latent trait score.

**Conclusions:**

Our findings did not support measurement invariance with respect to assessment method. Because of the small effect sizes, the measurement differences between the online assessed CAPE and its paper and pencil original can be neglected without major consequences for research purposes. However, a person with a high vulnerability for psychotic symptoms would score 4.80 points lower on the total scale if the CAPE is assessed online compared to paper and pencil assessment. Therefore, for clinical purposes, one should be cautious with online assessment of the CAPE.

## Introduction

Mounting evidence suggests that the level of psychosis varies continuously in the population; ranging from normal functioning to transitory subclinical psychotic experiences, to clinical diagnosis [1], [2]. Subclinical psychotic experiences in the general population have a prevalence of 17,5% [3], [4]. In about 8% of the population, the symptoms persist and eventually develop into a clinical psychosis [5]. Frequent use of cannabis and alcohol abuse are associated with an increased prevalence of subclinical psychosis [1]. Provided that transitions over the psychotic continuum occur [5], assessment of sub-threshold psychotic experiences in the general population is of importance. The majority of the studies investigating psychotic symptoms in the general population use self-report questionnaires, although information about reliability and validity is scarce. A proved reliable and valid instrument for the self-report of psychotic experiences in the general population is the Community Assessment of Psychic Experiences (CAPE) [6], [7]. The CAPE has also been shown to be a useful screening tool for first episode psychosis in clinical samples [8], [9].

A fast and cost effective method for the assessment of large study populations is online assessment. In the last decade the use of online questionnaires in large epidemiological studies has increased rapidly. Assessment by web based questionnaires has several advantages over the use of paper and pencil questionnaires. Compared to paper and pencil, online assessments are less time consuming, less costly, and provide an easy access to large populations [10], [11]. Online data entry is automated, and therefore less sensitive to entry errors and missing data than manually entered data [12], [13]. In addition, research participants have reported a preference in favour of online completion of a questionnaire [14], [15]. Although these are promising results, administrating questionnaires online also has its own limitations. For instance, variation in speed of internet connection may cause variation in the duration of test completion; and termination of test sessions may occur by loss of internet connection. Furthermore, the test appearance may be inconsistent because of variation in screen size and screen resolution [16]. In addition, we have to take into consideration the possibility that psychometric properties of an online test are not necessarily identical to those of a paper and pencil test, even if the online version is a direct translation of the original [17], [18].

The factor structure of a paper and pencil instrument may change when the instrument is translated into an online version [19], [20]. For example, a person who completes a questionnaire by internet may be more likely to respond positively to sensitive health items in the perceived anonymity before the screen, compared to a person who completes a paper and pencil version which will be manually checked by the researcher. Although several studies report that the online version of an instrument was equally reliable and valid as the paper and pencil version [12], [21]–[23], differences in factor structure have also been reported. For instance, previous studies showed differences in the factor structure of internet questionnaires compared to the paper and pencil equivalents [20], [24], [25]. Also, systematically different responses were obtained when a personality questionnaire was completed online [22], [26].

Measurement theory assumes that an instrument (e.g., a questionnaire) has been developed in order to assess an underlying latent trait that cannot be directly observed. The instrument typically includes multiple directly observed variables (e.g., test items) which are indicators of the latent trait of interest. The response, or observed score, on each test item represents the sum of i) the weighted unobserved latent trait scores and ii) measurement error. A factor model is a representation of a set of linear regression relations between the items and one or more latent traits, e.g. latent factors [10]. The strength of the linear relation between each factor and an associated item is referred to as the factor loading [10]. The factor model is an essential part of the assessment of latent traits.

Differences in factor structure imply that instruments do not measure the same construct and cannot be treated as equal or comparable to each other [17]. In contrast, equivalent factor structures imply that mean differences in observed scores can be interpreted in terms of mean differences in the underlying latent factors, a concept referred to in the literature as measurement invariance (MI). MI implies that the response of a given person can be expected to depend on his or her score on the latent trait dimension, and not on other individual characteristics [27]. In the context of the present study, in which we aim to investigate whether items are measurement invariant with respect to online vs. paper and pencil assessment, an example of violation of MI is the hypothetical situation in which two persons with similar scores on a latent trait dimension have systematically different probabilities of responding positively to an item and therefore do not have similar scores on the observed item.

The aim of this study is to test whether the CAPE rating scale is measurement invariant with respect to assessment method i.e., online vs. paper and pencil. Internet data were collected by the Cannabis Quest study of the University Medical Centre of Utrecht [28], [29]. Paper and pencil data were collected by the Maastricht University Medical Centre. The factor structure of the internet and paper and pencil CAPE will be compared by testing for MI within a multigroup confirmatory factor analysis (MGCFA). MGCFA is a powerful method for analysing Measurement Invariance [30]. MGCFA permits a direct examination of measurement invariance by varying constraints across a series of nested models [31]. In addition, we will determine the effect sizes of any violation of MI as suggested by Meade [32].

## Method

### Participants

We used two separate samples of participants. Both samples were recruited in order to assess subclinical psychotic experiences in the general population. The first sample includes 21,838 Dutch-speaking participants, recruited between 2006 and 2009 by the Cannabis Quest study of the University Medical Centre (UMC) of Utrecht, the Netherlands, approved by the Medical Ethical Test Committee of the UMC Utrecht, reference number 06/100 [28]. The Cannabis Quest study investigates the relationship between cannabis use and subclinical psychiatric experiences in the general population. Participants were included in this study, irrespective of their level of cannabis use. We will refer to this sample as the “Internet sample”. In the Internet sample, subclinical psychosis was assessed by an online version of the CAPE self-report questionnaire using a publicly accessible project website. Participants were recruited by advertisement in cooperation with more than 100 colleges, universities and youth centres. We included participants between 10 and 60 years old who provided informed consent. To protect against random answers, participants who failed to correctly fill out two verification questions were excluded. After exclusion, 21,590 (83.2%) of the participants remained. Because of the administration by internet, there were no missing items in the CAPE assessment.

The second sample comprised 805 Dutch speaking participants, recruited from a non-clinical general population in the city of Sittard by the Maastricht University Medical Centre (MUMC), Maastricht, the Netherlands [33], [34], approved by the Medical Ethical Test Committee of the MUMC. Within the municipality of Sittard, participants randomly received a letter in which they were asked to participate. Participants between 18 and 70 years old who provided written informed consent were included. The participants completed a paper and pencil version of the CAPE self-report questionnaire and the sample will be referred to as “the Paper sample”. Assessment was administered by self completion in the presence of a research assistant, at home or at the MUMC. Nine participants with more than 4 (10%) missing items were excluded from subsequent analyses. In the remaining sample, n = 796, missing item-scores were coded as missing (−1) and were treated as such in the statistical analyses. The paper sample had a mean percentage of missingness of 0.01% with a maximum of 7.14% per subject. The mean missingness per item was 0.03 with a maximum of 9 missing scores (1.13%) for item 2.

### Measures

The Community Assessment of Psychic Experiences (CAPE) [6] measures psychotic experiences in the general population through a 42 item self-report questionnaire. The items measure symptomatology in 3 domains: Positive Symptoms (20 items), Negative Symptoms (14 items) and Depression Symptoms (8 items). Each item is rated at a 4 point Likert scale from 1 to 4 for both symptom frequency and the degree of distress experienced due to the symptom. In both the Internet and Paper samples the Dutch version of the CAPE [7] was completed by the participants. For the analyses in the current study we used the frequency ratings only, as these are more widely used in previous studies. A further advantage of the frequency scores is that these are assessed in all participants while the degree of distress is only assessed in those participants in whom the symptom is present. To avoid response categories with very low endorsement rates, item categories with response rate frequencies below 5% were merged with the preceding category. This was equally applied in both samples. As a result, in both samples, items 5, 7, 14, 17, 23, 28, 30, 31, 33, 34, 35, and 41 where recoded into dichotomous items. The remaining items were recoded into items with 3 response categories. After recoding, the total CAPE score ranges between a minimum of 42 and a maximum of 114 points. Table S1 shows the response categories for each CAPE item and the response rate for each category in percentages.

### Statistical analyses

#### Measurement Invariance analyses

To test for measurement invariance, the CAPE item scores of both samples will be compared within a multigroup confirmatory factor analysis by use of the Theta parameterization [35]. We used the WLSMV estimator for non-normally distributed data in Mplus for statistical analysis with latent variables Version 5.1 [36]. Goodness of model fit of the baseline model (i.e., the model which imposes least constraints to the similarity of the factor structures) will be determined by the Comparative Fit Index (CFI), the Tucker Lewis Index (TLI), and the Root Mean Square Error of Approximation (RMSEA) [37], [38]. These indexes compare the observed sample covariance matrices with the estimated covariance matrices of the factor model. A CFI and TLI above 0.95 and a RMSEA below 0.05 indicate good model fit. An acceptable model fit is indicated by a CFI and TLI between 0.90 and 0.95 and a RMSEA value between 0.05 and 0.08 [39], [40]. The RMSEA has the advantage that it performs well with categorical data and is reasonably insensitive for the number of observations in the sample [39], [41]. After establishing goodness of fit of the baseline model, we will imply increasingly stringent model constraints to test the remaining levels of measurement invariance. A non-significant decrease in model fit in a more restricted model indicates measurement invariance of the factor models. The goodness of fit of nested models is evaluated by hierarchic likelihood ratio (χ^2^) tests. Specifically, the χ^2^ statistic is computed by taking twice the difference between the log-likelihood of the full model and the log-likelihood of a reduced model. The associated number of degrees of freedom is computed by Mplus as the difference in the degrees of freedom between the two hierarchic models. Note that for the WLSMV estimator, a standard chi- square difference test is not valid. The difference in chi-square values for two nested models using the WLSMV is not distributed as chi-square. Therefore, Mplus uses a two-step procedure to obtain a correct chi-square difference test. In the first step, the H1 model is estimated and the derivatives needed for the chi-square difference test are saved. In the second step, the H0 model is estimated and the chi-square difference test is computed using the derivatives from the H0 and H1 analyses. For a more detailed description of the Mplus chi square difference testing we refer to Asparouhov and colleagues [42].

As a baseline model, we used the 3-factor model described by Stefanis and colleagues [6], confirmed by Brenner and colleagues [43]. The baseline model will be fit to both samples simultaneously within a Multigroup Confirmatory Factor Analysis. If the model provides an acceptable fit in both samples, the internet and paper and pencil versions show *Configural Invariance*. Configural Invariance implies that in both samples the CAPE items load on the same factors.

The second step in the MI analysis, is to test for *Metric Invariance* [44]. Metric Invariance is investigated by constraining the factor loadings of the 3-factor model to be equal between the Internet sample and Paper sample. Together with the test for Configural Invariance, the test for Metric Invariance is considered to be the most important test of MI [27], [45].

In the final step of the MI we will test for *Strict Factorial Invariance* by including an equality constraint to the residual variances of the observed responses. This way we test whether the measurement accuracy is equal between the two samples. Only when all three levels of MI are confirmed, differences in observed scores can be interpreted as differences in unobserved latent scores.

### Effect size indices

According to Meade [32] violation of MI could best be considered as a continuum rather than a dichotomous ‘invariant’ or ‘not invariant’. For instance, statistically significant violation of MI may have little clinical relevance. Calculation of effect size indices allow researchers to decide whether they wish to alter the measure in some way, ignore the MI, or correct observed score differences [32]. Therefore, in addition to formal tests for MI, we will calculate effect sizes by estimating the Signed Item Difference In the Sample (SIDS) index and the Signed Test Difference in the Sample (STDS) [32].

In order to determine the SIDS and STDS we first have to compute for each sample the average expectancy score (ES) for all 42 CAPE items based on the specific model parameters [32]. For any value of the latent trait score, the ES can be computed as the sum of the probabilities of a response to each of the response options, i.e. answer categories of the item, multiplied by the value of that response option. The ES is similar to an item-level true score and has a range from the lowest to the highest response option [32]. In the current analysis we will determine for a person with a latent score of 2 standard deviations above average (i.e., a clinically vulnerable subject), the ES for each CAPE item assessed by Internet or Paper and Pencil. Individuals with lower latent trait scores are expected to have a lower ES and in consequence, a lower effect size index.

The SIDS index will be computed as the difference in ESs across the Internet sample, compared to the ESs of the Paper sample. The SIDS is in the same metric as the observed scores. This makes it possible to interpret the effect of MI on observed means in a sample in an easy way [32]. For instance, a SIDS of −1.5 for a CAPE item with 3 response options implies that Internet sample members would be expected to score 1.5 points lower on the item than would Paper sample members with the same latent trait scores.

The STDS index is the sum of the SIDS indices. A STDS of −2.5 indicates that on average Internet sample members would be expected to score 2.5 points lower on the summed scale compared to members of the Paper sample. This difference would only be present in case of violation of MI [32].

Effect size analyses will be calculated by use of the statistical package R, version 2.15.1 [46].

### Application of a cut-off score for clinical vulnerability

Boonstra et al. [8] determined a cut-off score, based on paper and pencil assessment, to improve recognition of first episode psychosis in first contact with mental health care services. The authors showed that a score of 50 points or higher on the frequency dimension of the CAPE positive symptom items, provided the most optimal sensitivity of 77% and specificity of 70.5%. For both samples, we will determine the proportion of participants scoring above this cut-off.

## Results

### Participants

The final sample includes 22,386 participants: 796 participants of the Paper Sample and 21,590 participants of the Internet Sample. Gender is not equally distributed between samples. The percentage of males is significantly higher in the Internet sample (50.6%) than in the paper sample (38.4%) (χ^2^ (1) = 45.8, P<0.001). For two participants in the Paper sample, gender was unknown.

The mean age in the internet sample (23.4 years; SD = 12.3) is significantly lower than the mean age in the paper sample (44.36 years; SD = 12.5) (t (854.1) = 46.6, p<.001). Members of the Paper sample have on average a lower total CAPE score in comparison with the Internet sample; t (860.6) = −10.3, P<0.001. This difference is accounted for by differences in negative symptoms and positive symptoms. There is no significant difference in depression symptoms. A summary of sample characteristics can be viewed in Table 1.

**Table 1:** Demographic characteristics, mean CAPE scores, and cut-off scores, of the Paper and Internet sample.

	*Internet Sample*	*Paper Sample*
	(N = 21590)	(N = 796)
**Mean (SD) Age in years**	23.4 (12.3)	44.36 (12.5)
**Gender (% female)**	49.4%*	61.4%*
**Mean (SD) Total CAPE symptom score**	64.6 (10.0)*	61.0 (9.6)*
**Mean (SD) Depression symptom score**	13.5 (2.7)	13.5 (2.8)
**Mean (SD) Negative symptom score**	23.3 (4.9)*	22.4 (3.9)*
**Mean (SD) Positive symptom score**	27.7 (4.5)*	25.0 (4.7)*
**Number of Subjects (%) with positive symptoms >50**	16 (0.07)	0 (0)

*Note: SD = Standard Deviation.*

* Value differs between Internet and Paper samples at significance level P<0.001.

### Measurement invariance analyses

Because of computational problems we could not estimate both the factor variances and the residual variances in both groups in the baseline model. Therefore, in the baseline model we equated the residual variances to be equal across groups. Subsequently, we estimated the residual covariances for the first and second step of the MI analyses.

For improvement of model fit we added a correlation between the residual items of items 13, ‘being special’, and 11, ‘being important’ and items 15, ‘Telepathy’ and 20, ‘Voodoo’, to the model. These items showed a high correlation, indicating that adding these parameters to the model improved model fit.

A Confirmatory Factor Analysis, conducted for both samples separately provided an acceptable fit to the data according the RMSEA and TLI (Paper sample; RMSEA = 0.05, TLI = 0.95) (Internet sample; RMSEA = 0.05, TLI = 0.94). However, the CFI did not indicate an acceptable fit (CFI = 0.86 and 0.80 for the Paper and Internet Sample respectively). While two of the three fit indices indicate an acceptable fit, this model was retained as the baseline model. Configural Invariance between the Internet Sample and the Paper Sample was confirmed as the multi group 3-factor baseline model provided an acceptable fit according to the RMSEA and TLI (RMSEA = 0.05,TLI = 0.94) even though the CFI value again did not indicate an acceptable fit (CFI = 0.81). The internet and paper samples are similar with respect to the number of factors and the configuration of the factor loadings (i.e., the three factors load on the same observed items).

Metric Invariance (i.e., equality of factor loadings) was not confirmed. According to the χ^2^ difference test, the restricted model showed a significantly worse fit compared to the less restricted Configural Invariance model (χ^2^ (23) = 152.75, p<0.001). Overall, the Paper sample showed higher item loadings on the three latent factors compared to the Internet sample. Because Metric Invariance was not confirmed, we will not report the results of the test for Strict Factorial Invariance here but these results are included in an overview of the CFA and MI results presented in Table 2 and Table 3. Table 4 shows an overview of the factor loadings of both samples in the Configural Invariance model.

**Table 2:** Free parameters and Fit indices CFA analyses total Internet and Paper sample.

CFA Analysis	Number of Free Parameters	χ^2^ (df)	RMSEA	CFI	TLI
**Paper**	**119 free parameters;**	**629.17 (185)** **	**0.05**	**0.86**	**0.95**
	42 factor loadings				
	72 thresholds				
	3 factor covariances				
	2 residual correlations				
**Internet**	**119 free parameters;**	**30168.35 (506)** **	**0.05**	**0.8**	**0.94**
	42 factor loadings				
	72 thresholds				
	3 factor covariances				
	2 residual correlations				

*Note: CFA = Confirmatory Factor Analysis. df = degrees of freedom.*

** *p<0.001.*

**Table 3:** Free parameters and Fit indices MI analyses total Internet and Paper sample.

MI Analysis	Nr. of Free Parameters	χ^2^ (df)	RMSEA	CFI	TLI	χ^2^ difftest^a^
**Configural model**	**238 free parameters;**	**17999.62 (556)** **	**0.05**	**0.81**	**0.94**	
***Group 1 paper***	42 factor loadings					
	72 thresholds					
	2 residual correlations					
	3 factor covariances					
***Group 2 Internet***	42 factor loadings					
	72 thresholds					
	2 residual correlations					
	3 factor covariances					
**Metric invariance**	**197 free parameters;**	**15462.1 (502)** **	**0.05**	**0.84**	**0.94**	**152.75 (23)** **
***Group 1 paper***	42 factor loadings					
	72 thresholds					
	2 residual correlations					
	3 factor covariances					
***Group 2 Internet***	27 thresholds					
	6 factor (co)variances					
	3 factor means					
	42 residual variances					
**Strong invariance**	**170 free parameters;**	**15801.84 (503)** **	**0.05**	**0.83**	**0.94**	**1299.87 (23)** **
***Group 1 paper***	42 factor loadings					
	72 thresholds					
	2 residual correlations					
	3 factor covariances					
***Group 2 Internet***	6 factor (co)variances					
	3 factor means					
	42 residual variances					
**Strict invariance**	**128 free parameters;**	**10998.13 (386)** **	**0.05**	**0.88**	**0.95**	**131.1 (34)** **
***Group 1 paper***	42 factor loadings					
	72 thresholds					
	2 residual correlations					
	3 factor covariances					
***Group 2 Internet***	6 factor (co)variances					
	3 factor means					

*Note: MI = Measurement Invariance. df = degrees of freedom.*

*Metric invariance; model fit compared to fit configural model. Strong invariance; model fit compared to fit Metric invariance model. Strict invariance; model fit compared to fit Strong invariance model.*

a *χ^2^ difftest was conducted in Mplus by use of WLSMV estimator.*

** *p<0.001.*

**Table 4:** Factor loadings, SIDS and STDS of the least restricted 3 factor model of the measurement invariance analysis for categorical data, **STDS = −4.80**.

CAPE	Internet sample	Paper sample	SIDS	STDS
**Factor 1 (Depression)**	**Factor loadings (s.e.)**	**Factor loadings (s.e.)**		**−1,37**
*Item 1 Sad*	**0.99 (0.02)**	**1.41 (0.10)**	**−0.21**	
*Item 9 Pessimism*	**0.93 (0.01)**	**1.30 (0.08)**	**−0.23**	
*Item 12 No future*	**1.21 (0.02)**	**1.37 (0.13)**	**−0.13**	
*Item 14 Not worth living*	**1.23 (0.02)**	**1.31 (0.13)**	**−0.02**	
*Item 19 Frequency cry*	**0.37 (0.01)**	**0.50 (0.05)**	**−0.19**	
*Item 38 Guilty*	**0.68 (0.01)**	**1.09 (0.08)**	**−0.35**	
*Item 39 Failure*	**1.33 (0.02)**	**1.43 (0.11)**	**−0.06**	
*Item 40 Feeling tense*	**0.73 (0.01)**	**1.15 (0.08)**	**−0.18**	
**Factor 2 Positive Symptoms**				**−3.29**
*Item 2 Double meaning*	**0.71 (0.01)**	**0.81 (0.07)**	**−0.07**	
*Item 5 Messages from TV*	**0.52 (0.01)**	**0.67 (0.08)**	**−0.07**	
*Item 6 False appearance*	**0.67 (0.01)**	**0.63 (0.06)**	**0.02**	
*Item 7 Being persecuted*	**0.79 (0.02)**	**0.90 (0.13)**	**−0.05**	
*Item 10 Conspiracy*	**1.02 (0.02)**	**1.26 (0.16)**	**−0.22**	
*Item 11 Being important*	**0.32 (0.01)**	**0.52 (0.08)**	**−0.21**	
*Item 13 Being special*	**0.36 (0.01)**	**0.43 (0.06)**	**−0.07**	
*Item 15 Telepathy*	**0.43 (0.01)**	**0.50 (0.05)**	**−0.07**	
*Item 17 influenced by devices*	**0.53 (0.01)**	**0.50 (0.10)**	**0.01**	
*Item 20 Voodoo*	**0.41 (0.01)**	**0.49 (0.06)**	**−0.08**	
*Item 22 Odd looks*	**0.54 (0.01)**	**0.82 (0.09)**	**−0.35**	
*Item 24 Thought withdrawal*	**0.90 (0.02)**	**1.46 (0.22)**	**−0.56**	
*Item 26 Thought insertion*	**0.99 (0.02)**	**1.23 (0.14)**	**−0.23**	
*Item 28 Thought broadcasting*	**0.70 (0.02)**	**1.07 (0.14)**	**−0.16**	
*Item 30 Thought echo*	**0.72 (0.02)**	**0.93 (0.12)**	**−0.09**	
*Item 31 External control*	**1.02 (0.02)**	**1.36 (0.20)**	**−0.15**	
*Item 33 Verbal hallucinations*	**0.99 (0.02)**	**1.02 (0.19)**	**−0.01**	
*Item 34 Voices conversing*	**1.16 (0.04)**	**1.99 (1.02)**	**−0.37**	
*Item 41 Capgras*	**0.95 (0.03)**	**1.17 (0.29)**	**−0.10**	
*Item 42 Visual hallucinations*	**0.76 (0.02)**	**1.19 (0.18)**	**−0.46**	
**Factor 3 Negative Symptoms**				**−0.14**
*Item 3 Lack of enthusiasm*	**0.90 (0.01)**	**0.95 (0.07)**	**−0.01**	
*Item 4 Not talkative*	**0.57 (0.01)**	**0.58 (0.05)**	**−0.01**	
*Item 8 No emotion*	**0.67 (0.01)**	**0.48 (0.05)**	**0.18**	
*Item 16 No interest in others*	**0.58 (0.01)**	**0.68 (0.06)**	**−0.07**	
*Item 18 lack of motivation*	**0.87 (0.01)**	**1.05 (0.07)**	**−0.08**	
*Item 21 No energy*	**0.74 (0.01)**	**0.91 (0.07)**	**−0.10**	
*Item 23 Empty mind*	**0.46 (0.01)**	**0.59 (0.06)**	**−0.06**	
*Item 25 Lack of activity*	**0.77 (0.01)**	**1.05 (0.08)**	**−0.17**	
*Item 27 Blunted feelings*	**0.98 (0.01)**	**0.85 (0.07)**	**0.12**	
*Item 29 Lack of spontaneity*	**0.76 (0.01)**	**0.73 (0.06)**	**0.03**	
*Item 32 Blunted emotions*	**1.02 (0.02)**	**1.02 (0.08)**	**0.00**	
*Item 35 Lack of hygiene*	**0.71 (0.01)**	**0.70 (0.08)**	**0.00**	
*Item 36 Unable to terminate*	**0.78 (0.01)**	**0.74 (0.06)**	**0.03**	
*Item 37 Lack of hobby*	**0.76 (0.01)**	**0.76 (0.07)**	**0.00**	

In order to verify that the model scaling was done correctly, we repeated the analyses by use of the Delta parameterisation. The MI analyses in Delta parameterisation provided highly similar results and confirmed violation of Metric invariance (data not shown).

### Effect size measurement

To estimate the extent to measurement invariance is violated, we computed the ES for each CAPE item assessed by Internet (ESi) or Paper and Pencil (ESp). The SIDS indices for the individual items and the STDS indices for each of the three factors are summarized in Table 4. The SIDS indices are small, with a range of 0 (items 35 and 37) to −0.56 (item 24) and an average SIDS of −0.11. The majority of the SIDS values are negative in line with the lower factor loadings in the Internet sample.

The STDS index for the total CAPE score is −4.80, thus on average, members from the Internet sample with a latent score of 2 would be expected to score 4.8 points lower on the total CAPE than would Paper sample members with the same latent trait score.

### Application of a cut off score for clinical vulnerability

Table 1 shows the proportion of participants with a score of 50 points or higher on the frequency dimension of the CAPE positive symptom items in both samples. No participants in the Paper sample met the cut-off, compared to sixteen participants (0.07%) in the Internet sample.

When we apply the cut-off on the Internet sample taking into account the fact that Internet assessment is associated with lower scores (i.e., STDS is −3.29 for the positive symptoms), we would expect that an additional n = 36 participants, 69.23%, would have met the cut-off if they would have completed the paper and pencil version. These participants had a total score between 47 and 50 on the CAPE positive symptoms scale completed by Internet.

### Post hoc analyses

To test whether the violation of Metric invariance is attributable to group differences in age and gender, we repeated the measurement invariance analysis with i) a subgroup of participants matched based on age and ii) a subgroup of participants matched based on gender. Data were matched by use of library ‘Matching: Multivariate and Propensity Score Matching with Balance Optimization’ [47] of the statistical package R version 2.15.0 [46].

### Age

The Internet and Paper samples matched for age each included N = 609 participants, with a mean age of 40.1 years for the Internet sample and 41.0 years for the Paper sample. Age was equally distributed between the two samples; t (1211.968) = −1.066, P = 0.3. Next, we compared the data of both samples matched for age within a multigroup confirmatory factor analysis. As in the larger total sample, factor loadings were higher in the Paper sample compared to the Internet sample (χ^2^ (25) = 69.55, p<0.001). Table S3 and Table S4 provide an overview of the CFA and MI results of the samples matched for age. Inspection of the factor loadings revealed that differences were similar to the differences found in the total sample. Table S2 shows an overview of the factor loadings in the Configural variance model.

In addition to the MI, the SIDS and STDS effect size indices were calculated for a person with a latent score of 2 within the Internet and Paper samples matched for age. The SIDS indices and STDS indices for each factor are summarized in Table S2. With a mean SIDS of −.0.08 and a STDS of −3.50, the samples matched for age showed similar effect sizes as the larger complete samples.

### Gender

The Internet and Paper samples matched for gender each included N = 793 participants, with gender identically distributed between samples, 38.5% male and 61.5% female. Next, we compared the data of both samples matched for gender within a multigroup confirmatory factor analysis. As in the larger total sample and in the sample matched for age, factor loadings were higher in the Paper sample compared to the Internet sample (χ^2^ (27) = 124.03, df = 27 p<0.001). Table S6 and Table S7 provide an overview of the CFA and MI results of the samples matched for gender. Inspection of the factor loadings revealed that differences were similar to the differences found in the large sample and in the age matched sample. Table S5 shows an overview of the factor loadings of both samples matched for gender in the Configural variance model of the measurement analysis.

SIDS and STDS effect size indices were calculated for a person with a latent score of 2 within the Internet and Paper samples matched for gender. The SIDS indices and STDS indices for each factor are summarized in Table S5. With a mean SIDS of −0.13 and a STDS of −5.47, the sex-matched samples showed similar effect sizes compared to the total samples suggesting that the different distribution of sex in the Internet and Paper and pencil samples was not responsible for the violation of measurement invariance.

## Discussion

In the present study, we aimed to investigate the equivalence in psychometric properties of an online assessed CAPE self-report instrument and the paper and pencil original. Data of two large samples, an Internet sample including 21,590 participants and a Paper sample including 796 participants, was analysed within a multi group confirmatory factor analysis framework.

### Measurement invariance in Internet vs. Paper and pencil versions of the CAPE

Our findings did not support measurement invariance with respect to assessment method (i.e., online vs. paper and pencil). Overall the CAPE items assessed on the Internet showed lower factor loading values in comparison with the original paper and pencil CAPE items. This implies that the latent variables, Depression, Positive Symptoms and Negative Symptoms [6], [7] of the online version have a weaker relation with the corresponding items than the paper and pencil version.

However, despite statistically significant violation of measurement invariance, the effect sizes were small. Analysis of effect size indices showed that the Internet sample had lower expected scores on the CAPE items compared to the Paper sample. For a subject with a latent score of 2 (i.e., this person would score 2 standard deviations above average), the expected sum score of online administration is 4.80 points lower compared to paper and pencil administration. As this concerns a relatively small difference at a total sum scale ranging from 42 to 114 with a standard deviation of 10.0, we argue that this difference can be neglected for research purposes. However, if the CAPE instrument is used for clinical reasons, e.g., guarding decisions with respect to referral for treatment, one has to be cautious interpreting online assessments.

Application of the cut-off of a clinical threshold of 50 as defined by Boonstra and colleagues [8] suggests that 69.23%, of the participants vulnerable for a psychotic disorder would not be detected using online administration. Therefore, if the aim is to select clinically vulnerable participants, one should be cautious with online assessment of the CAPE self-report questionnaire. By interpreting these results we have to take into consideration that for the current study we recoded the CAPE items from items with 4 response categories for symptom frequency in to items with 2 or 3 response categories. As a result the total STDS of −4.80 and the positive symptoms STDS of −3.29 would possibly be even larger for a person with a latent score of 2 when response category 4, the highest symptom frequency score, would be taken into account for the analysis.

The current study confirms the concerns [22], [25], [26] that have been raised with respect to internet administration of paper and pencil instruments. Test administrators should be cautious when using online administration as psychometric properties of paper and pencil tests are not necessarily similar compared to online tests [22]. As we have argued above, for the CAPE, these concerns are mostly limited to administration for clinical purposes. Clinical norm scores which have been developed based on paper and pencil administration should not be applied to online assessments without careful consideration of the implications.

### Post-hoc analyses to test for potential mediation by age and gender

Age and gender were not equally distributed in the Internet and Paper samples. Therefore, two post-hoc analyses were performed in which the MI analysis was repeated in samples that were matched based on age and gender, respectively. The results of these analyses confirmed the findings of the total Internet and Paper samples as Metric Invariance was significantly violated in these post-hoc analyses. This shows that the difference in factor structure between samples is not explained by age or gender differences between groups.

### Strengths and limitations

The main strength of this study is the inclusion of two large population-based samples allowing for formal testing of measurement invariance of the factor structure of the CAPE self report questionnaire.

The findings of this study should be considered in view of the following limitations. First, no information on cannabis use is available for the Paper sample. Therefore, we cannot rule out the possibility that the two samples differ in the frequency of cannabis use and we could not statistically control for the level of cannabis use in our analyses. However, both samples have been recruited in the general population and the majority of the Internet sample used no cannabis (27.05%) or used very low levels (38.19%). These percentages are comparable to the 26% average cannabis use in the Netherlands and 30.2%–43.1% in age group 16–18 years old (source: Trimbos Institute, Dutch Institute of Mental Health and Addiction). Therefore, we expect no major differences between samples with respect to demographic factors such as cannabis use. Second, in the Internet sample, we did not collect information on the specific device that was used to complete the questionnaire, e.g., the use of a smartphone vs. a personal computer. However, data have been collected between 2006 and 2009, and the use of smartphones was not yet substantial in the Netherlands in these years.

### Conclusion

Compared to paper and pencil administration, online administration of questionnaires has important advantages in large epidemiological studies. However, when observed scores are compared between samples that have used a different type of administration, it should first be tested whether the assessment of the underlying concepts is similar. We have tested measurement invariance of a questionnaire that is used to assess subclinical psychotic experiences (i.e., the CAPE) in a large sample including 22,386 participants. Our results show small but significant differences in the factor structure of the CAPE symptoms. For clinical purposes, e.g., the selection of participants at increased risk for psychosis, we advise to use paper and pencil administration. Alternatively, a novel clinical cut-off score could be developed based on data that have been collected online.

## Supporting Information

Table S1 **CAPE item categories response rate (RR) frequencies in % for the Paper sample and the Internet sample.**
(DOCX)

Table S2 **Factor loadings, SIDS and STDS of the least restricted 3 factor model of the measurement invariance analysis for categorical data of the Paper and Internet sample matched for age.** Total STDS = −3.5.
(DOCX)

Table S3 **Free parameters and Fit indices CFA analyses Internet and Paper sample matched for age.**
(DOC)

Table S4 **Free parameters and Fit indices MI analyses Internet and Paper sample matched for age.**
(DOC)

Table S5 **Factor loadings, SIDS and STDS of the least restricted 3 factor model of the measurement invariance analysis for categorical data of the Paper and Internet sample matched for gender.** Total STDS = −5.47.
(DOC)

Table S6 **Free parameters and Fit indices CFA analyses Internet and Paper sample matched for gender.**
(DOC)

Table S7 **Free parameters and Fit indices MI analyses Internet and Paper sample matched for gender.**
(DOC)

## References

[1] van Os J, Linscott RJ, Myin-Germeys I, Delespaul P, Krabbendam L (2009) A systematic review and meta-analysis of the psychosis continuum: evidence for a psychosis proneness-persistence-impairment model of psychotic disorder. Psychol Med 39: 179–195. 18606047 10.1017/S0033291708003814

[2] Cougnard A, Marcelis M, Myin-Germeys I, De Graaf R, Vollebergh W, et al (2007) Does normal developmental expression of psychosis combine with environmental risk to cause persistence of psychosis? A psychosis proneness-persistence model. Psychol Med 37: 513–527. 17288646 10.1017/S0033291706009731

[3] Spauwen J, Krabbendam L, Lieb R, Wittchen HU, van Os j (2003) Sex differences in psychosis: normal or pathological? Schiz Res 62: 45–49. 10.1016/s0920-9964(03)00063-x 12765742

[4] van Os J, Hanssen M, Bijl RV, Ravelli A (2000) Strauss (1969) revisited: a psychosis continuum in the general population? Schiz Res 45: 11–20. 10.1016/s0920-9964(99)00224-8 10978868

[5] Hanssen M, Bak M, Bijl R, Vollebergh W, van Os J (2005) The incidence and outcome of subclinical psychotic experiences in the general population. Br J Clin Psychol 44: 181–191. 16004653 10.1348/014466505X29611

[6] Stefanis NC, Hanssen M, Smirnis NK, Avramopoulos DA, Evdokimidis IK, et al (2002) Evidence that three dimensions of psychosis have a distribution in the general population. Psychol Med 32: 347–358. 11866327 10.1017/s0033291701005141

[7] Konings M, Bak M, Hanssen M, van Os J, Krabbendam L (2006) Validity and reliability of the CAPE: a self-report instrument for the measurement of psychotic experiences in the general population. Acta Psychiatr Scand 114: 55–61. 16774662 10.1111/j.1600-0447.2005.00741.x

[8] Boonstra N, Wunderink L, Sytema S, Wiersma D (2009) Improving detection of first-episode psychosis by mental health-care services using a self-report questionnaire. Early Interv Psychiatry 3: 289–295. 22642732 10.1111/j.1751-7893.2009.00147.x

[9] Mossaheb N, Becker J, Schaefer MR, Klier CM, Schloegelhofer M, et al (2012) The Community Assessment of Psychic Experience (CAPE) questionnaire as a screening-instrument in the detection of individuals at ultra-high risk for psychosis. Schiz Res 141: 210–214. 10.1016/j.schres.2012.08.008 22986044

[10] Bollen KA (1989) A new incremental fit index for general structural equation models. Sociological Methods & Research 16: 303–316.

[11] Musch J, Reips U (2000) A brief history of web experimenting. In:Birnbaum MH, editors. Psychological experiments on the internet. New York: Academic Press. pp. 61–78.

[12] Cronk BC, West JL (2002) Personality research on the Internet: a comparison of Web-based and traditional instruments in take-home and in-class settings. Behav Res Methods Instrum Comput 34: 177–180. 12109009 10.3758/bf03195440

[13] Kongsved SM, Basnov M, Holm-Christensen K, Hjollund NH (2007) Response rate and completeness of questionnaires: a randomized study of Internet versus paper-and-pencil versions. J Med Internet Res 9: e25. 17942387 10.2196/jmir.9.3.e25 PMC2047288

[14] Andersson G, Kaldo-Sandstrom V, Strom L, Stromgren T (2003) Internet administration of the Hospital Anxiety and Depression Scale in a sample of tinnitus patients. J Psychosom Res 55: 259–262. 12932800 10.1016/s0022-3999(02)00575-5

[15] Vispoel WP, Boo J, Bleiler T (2001) Computerized and paper-and-pencil versions of the Rosenberg Self- Esteem Scale. Educational and Psychological Measurement 61: 461–474.

[16] Bartram D (2006) Testing on the Internet: Issues, Challenges and Opportunities in the Field of Occupational Assessment. In Bartram D, Hambelton RK. Computer-Based Testing and the Internet: Issues and Advances. John Wiley & Sons, Ltd. pp. 13–37.

[17] Buchanan T (2003) Internet-based questionnaire assessment: appropriate use in clinical contexts. Cogn Behav Ther 32: 100–109. 16291542 10.1080/16506070310000957

[18] Buchanan T, Heffernan TM, Parrott AC, Ling J, Rodgers J, et al (2010) A short self-report measure of problems with executive function suitable for administration via the Internet. Behav Res Methods 42: 709–714. 20805593 10.3758/BRM.42.3.709

[19] Bartram D (2005) The Great Eight competencies: a criterion-centric approach to validation. J Appl Psychol 90: 1185–1203. 16316273 10.1037/0021-9010.90.6.1185

[20] Buchanan T, Ali T, Heffernan TM, Ling J, Parrott AC, et al (2005) Nonequivalence of on-line and paper-and-pencil psychological tests: the case of the prospective memory questionnaire. Behav Res Methods 37: 148–154. 16097355 10.3758/bf03206409

[21] Coles ME, Cook LM, Blake TR (2007) Assessing obsessive compulsive symptoms and cognitions on the internet: evidence for the comparability of paper and Internet administration. Behav Res Ther 45: 2232–2240. 17306222 10.1016/j.brat.2006.12.009

[22] Meade AW, Michels LC, Lautenschlager GJ (2007) Are internet and paper-and-pencil personality tests truly comparable? : An experimental design measurement invariance study. Organizational Research Methods 10: 322–345.

[23] Meyerson P, Tryon WW (2003) Validating internet research: a test of the psychometric equivalence of internet and in-person samples. Behav Res Methods Instrum Comput 35: 614–620. 14748506 10.3758/bf03195541

[24] Hannon R, Adams P, Harrington S, Fries-Dias C, Gipson MT (1995) Effects of Brain Injury and Age on Prospective Memory Self-Rating and Performance. Rehabilitation Psychology 40: 289–298.

[25] Wu RC, Thorpe K, Ross H, Micevski V, Marquez C, et al (2009) Comparing administration of questionnaires via the internet to pen-and-paper in patients with heart failure: randomized controlled trial. J Med Internet Res 11: e3. 19275979 10.2196/jmir.1106 PMC2762767

[26] Vecchione M, Alessandri G, Barbaranelli C (2012) Paper-and-Pencil and Web-Based Testing: The Measurement Invariance of the Big Five Personality Tests in Applied Settings. Assessment 19: 243–246. 21862530 10.1177/1073191111419091

[27] Meredith W (1993) Measurement Invariance, factor analysis and factorial invariance. Psychometrika 58: 525–543.

[28] Schubart CD, van Gastel WA, Breetvelt EJ, Beetz SL, Ophoff RA, et al (2010) Cannabis use at a young age is associated with psychotic experiences. Psychol Med 41: 1301–1310. 20925969 10.1017/S003329171000187X

[29] Schubart CD, Boks MP, Breetvelt EJ, van Gastel WA, Groenwold RH, et al (2011) Association between cannabis and psychiatric hospitalization. Acta Psychiatr Scand 123: 368–375. 21198455 10.1111/j.1600-0447.2010.01640.x

[30] Cheung GW, Rensvold RB (2002) Evaluating goodness-of-fit indexes for testing measurement invariance. Structural Equation Modeling 9: 233–255.

[31] Vandenberg RJ (2002) Toward a further understanding of and improvement in measurement invariance methods and procedures. Organizational Research Methods 5: 139–158.

[32] Meade AW (2010) A taxonomy of effect size measures for the differential functioning of items and scales. J Appl Psychol 95: 728–743. 20604592 10.1037/a0018966

[33] Hanssen M, Peeters F, Krabbendam L, Radstake S, Verdoux H, et al (2003) How Psychotic are individuals with non-psychotic disorders? Soc Psychiatry Psychiatr Epidemiol 38: 149–154. 12616313 10.1007/s00127-003-0622-7

[34] Thewissen V, Bentall RP, Lecomte T, van Os J, Myin-Germeys I (2008) Fluctuations in self-esteem and paranoia in the context of daily life. J of Abn Psychol 1: 143–153. 10.1037/0021-843X.117.1.143 18266492

[35] Muthen B, Asparouhov T (2002) Latent Variable Analysis With Categorical, Outcomes: Multiple-Group And Growth Modeling In Mplus. Mplus Web Notes4: 1–22.

[36] Muthén LK, Muthén BO (1998–2010) Mplus User's Guide, 6 edn. Los Angeles, CA: Muthén, Muthén. 752 p.

[37] Bentler PM (1990) Comparative fit indexes in structural models. Psychol Bull 107: 238–246. 2320703 10.1037/0033-2909.107.2.238

[38] Bollen K, Long JS (1993) Testing structural equation models. Newbury Park, CA: Sage Publications Inc. 308 p.

[39] Hu LT, Bentler PM (1995) Evaluating model fit. In: Hoyle RH. Structural Equation Modeling. Concepts, Issues, and Applications. London: Sage. pp. 76–99.

[40] Browne MW, Cudeck R (1993) Alternative ways of assessing model fit. In:Bollen K, Long J.S. In Testing structural equation models. Newbury Park, CA: Sage Publications, Inc.pp. 136–162.

[41] Schermelleh-Engel K, Moosbrugger H, Müller H (2003) Evaluating the fit of structural equation models: Tests of significance and descriptive goodness-of-fit measures. Methods of Psychological Research Online 8: 23–74.

[42] Asparouhov T, Muthen B, Muthen L (2006) Robust Chi-Square Difference Testing with Mean and Variance Adjusted Test Statistics. Mplus Web Notes 10: 1–6.

[43] Brenner K, Schmitz N, Pawliuk N, Fathalli F, Joober R, et al (2007) Validation of the English and French versions of the Community Assessment of Psychic Experiences (CAPE) with a Montreal community sample. Schizophr Res 95: 86–95. 17693059 10.1016/j.schres.2007.06.017

[44] Horn JL, McArdle JJ (1992) A practical and theoretical guide to measurement invariance in aging research. Exp Aging Res 18: 117–144. 1459160 10.1080/03610739208253916

[45] Meade AW, Lautenschlager GJ (2004) A Monte-Carlo study of confirmatory factor analytic tests of measurement equivalence/invariance. Structural Equation Modeling 11: 60–72.

[46] R Development Core Team (2012) R: A language and Environment for Statistical Computing [2.15.1] R Foundation for Statistical Computing. Vienna, Austria.

[47] Sekhon JS (2011) Multivariate and Propensity Score Matching Software with Automated Balance Optimization: the Matching Package for R. Journal of Statistical Software 42: 1–52.

