# Supplementary material for: Does Assessment Type Matter? A Measurement Invariance Analysis of Online and Paper and Pencil Assessment of the Community Assessment of Psychic Experiences (CAPE)
Source: PLoS One. 2014 Jan 22;9(1):e84011. doi: 10.1371/journal.pone.0084011 (PMC3898946; doi:10.1371/journal.pone.0084011)
Supplement: Table S1 — CAPE item categories response rate (RR) frequencies in % for the Paper sample and the Internet sample. (DOCX) [file pone.0084011.s001.docx]

| **Table S1. CAPE item categories response rate (RR) frequencies in % for the Paper sample and the Internet sample.** | | | | | | | | | | | | |
| --- | --- | --- | --- | --- | --- | --- | --- | --- | --- | --- | --- | --- |
| **Item** | **% RR category 1** | | | **% RR category 2** | | | **% RR category 3** | | | **% RR category 4** | | |
|  | **Paper** | **Internet** | | **Paper** | **Internet** | | **Paper** | **Internet** | | **Paper** | **Internet** | |
| **1** | 11.4 | | 13.5 | 77.0 | | 77.3 | 11.1 | | 8.2 | 0.3 | | **1.0** |
| **2** | 39.4 | | 32.8 | 53.6 | | 58.3 | 5.5 | | 8.2 | **0.3** | | **0.8** |
| **3** | 52.3 | | 47.6 | 39.2 | | 42.0 | 6.8 | | 8.7 | 1.1 | | **1.7** |
| **4** | 38.8 | | 36.4 | 48.4 | | 48.0 | 11.4 | | 12.9 | 1.4 | | **2.8** |
| **5 **** | 70.9 | | 69.4 | 26.3 | | 27.1 | **2.4** | | **3.0** | **0.4** | | **0.5** |
| **6** | 12.9 | | 17.5 | 62.8 | | 56.8 | 22.4 | | 23.1 | **1.5** | | **2.6** |
| **7 **** | 89.7 | | 77.0 | 9.2 | | 20.3 | **0.8** | | **2.1** | **0.3** | | **0.6** |
| **8** | 59.9 | | 53.3 | 33.2 | | 35.4 | 5.8 | | 9.0 | **1.0** | | **2.3** |
| **9** | 32.4 | | 42.9 | 57.5 | | 48.6 | 8.9 | | 7.1 | 1.0 | | **1.4** |
| **10** | 83.4 | | 77.0 | 10.7 | | 17.9 | 1.4 | | 4.5 | **0.4** | | **0.6** |
| **11** | 87.7 | | 52.0 | 12.4 | | 33.7 | 1.0 | | 10.3 | **0.9** | | **4.1** |
| **12** | 76.9 | | 70.3 | 19.6 | | 23.9 | 2.8 | | 4.3 | 0.6 | | **1.5** |
| **13** | 70.9 | | 40.9 | 23.5 | | 42.1 | 4.6 | | 12.3 | **1.0** | | **4.7** |
| **14 **** | 78.4 | | 75.3 | 19.0 | | 21.3 | **1.8** | | **2.4** | **0.8** | | **0.9** |
| **15** | 38.3 | | 51.1 | 49.9 | | 38.5 | 9.8 | | 8.1 | **1.4** | | **2.3** |
| **16** | 19.3 | | 19.4 | 70.5 | | 67.5 | 8.9 | | 11.6 | **1.3** | | **1.5** |
| **17 **** | 92.0 | | 83.0 | 6.7 | | 12.6 | **0.9** | | **3.5** | **0.4** | | **0.9** |
| **18** | 24.6 | | 17.5 | 64.9 | | 59.2 | 9.5 | | 19.9 | 0.9 | | **3.4** |
| **19** | 52.8 | | 52.0 | 41.6 | | 42.3 | 5.2 | | 5.2 | **0.3** | | **0.5** |
| **20** | 63.7 | | 54.6 | 25.6 | | 31.6 | 6.3 | | 7.4 | 4.0 | | **0.5** |
| **21** | 15.6 | | 18.1 | 57.2 | | 62.3 | 22.4 | | 16.6 | 4.9 | | **3.1** |
| **22** | 72.2 | | 16.2 | 22.2 | | 55.7 | 4.0 | | 23.1 | **1.3** | | **5.1** |
| **23 **** | 57.4 | | 60.6 | 37.8 | | 34.6 | **4.5** | | **3.6** | **0.3** | | **1.1** |
| **24** | 87.1 | | 84.1 | 7.2 | | 10.3 | 5.5 | | 4.4 | **0.3** | | **0.5** |
| **25** | 56.9 | | 33.2 | 37.2 | | 51.5 | 5.2 | | 13.3 | **0.6** | | **2.0** |
| **26** | 88.2 | | 81.2 | 6.0 | | 14.1 | 5.5 | | 3.6 | **0.3** | | **1.5** |
| **27** | 58.3 | | 64.6 | 36.6 | | 29.4 | 4.4 | | **4.9** | **0.5** | | **1.1** |
| **28 **** | 90.3 | | 81.4 | 8.3 | | 15.9 | 1.3 | | **2.3** | **0.1** | | **0.5** |
| **29** | 44.0 | | 46.7 | 47.5 | | 43.4 | 7.3 | | 8.4 | 1.1 | | **1.6** |
| **30 **** | 88.4 | | 80.1 | 9.7 | | 16.5 | **1.8** | | **2.8** | **0.1** | | **0.6** |
| **31 **** | 90.8 | | 86.6 | 7.7 | | 10.5 | **1.0** | | **1.8** | **0.5** | | **1.1** |
| **32** | 60.6 | | 64.9 | 33.8 | | 28.3 | 4.8 | | 5.2 | **0.6** | | **1.6** |
| **33**** | 95.2 | | 89.3 | 3.9 | | 9.1 | **0.6** | | **1.1** | **0.1** | | **0.5** |
| **34 **** | 93.6 | | 95.7 | 6.0 | | 3.4 | **0.3** | | **0.6** | **0.0** | | **0.3** |
| **35 **** | 78.4 | | 70.0 | 21.0 | | 26.9 | **0.6** | | **2.3** | **0.0** | | **0.8** |
| **36** | 37.6 | | 30.8 | 48.9 | | 55.0 | 11.9 | | 11.9 | **1.4** | | **2.3** |
| **37** | 68.8 | | 58.6 | 73.1 | | 29.6 | 14.2 | | 8.8 | **2.1** | | **3.0** |
| **38** | 10.3 | | 8.6 | 73.1 | | 75.0 | 14.2 | | 14.2 | **2,1** | | **2.2** |
| **39** | 58.4 | | 52.4 | 35.4 | | 39.4 | 4.6 | | 6.0 | **1.1** | | **2.2** |
| **40** | **4.6** | | 8.7 | 68.1 | | 67.4 | 23.4 | | 20.1 | **3.8** | | **3.8** |
| **41 **** | 97.2 | | 95.4 | 2.3 | | 3.6 | **0.5** | | **0.6** | **0.0** | | **0.4** |
| **42** | 93.7 | | 87.0 | 5.5 | | 7.2 | 0.6 | | 5.1 | **0.1** | | **0.7** |

*Note: RR = response rate. Item categories with a response rate below 5% are merged within the preceding category. All items were re-coded in to items with 3 response rate frequencies 1-3. **items were re-coded in 2 categories 1-2.*
