# Supplementary material for: Does Assessment Type Matter? A Measurement Invariance Analysis of Online and Paper and Pencil Assessment of the Community Assessment of Psychic Experiences (CAPE)
Source: PLoS One. 2014 Jan 22;9(1):e84011. doi: 10.1371/journal.pone.0084011 (PMC3898946; doi:10.1371/journal.pone.0084011)
Supplement: Table S2 — Factor loadings, SIDS and STDS of the least restricted 3 factor model of the measurement invariance analysis for categorical data of the Paper and Internet sample matched for age. Total STDS = −3.5. (DOCX) [file pone.0084011.s002.docx]

**Table S2.** Factor loadings, SIDS and STDS of the least restricted 3 factor model of the measurement invariance analysis for categorical data of the Paper and Internet sample matched for age. **Total STDS =-3.5.**

| **CAPE** | **Internet sample** | **Paper sample** | **SIDS** | **STDS** |
| --- | --- | --- | --- | --- |
| **Factor 1 (Depression)** | Factor loadings (s.e.) | Factor loadings (s.e.) |  | -1.13 |
| *Item 1 Sad* | 1.03 (0.09) | 1.46 (0.12) | -0.20 |  |
| *Item 9 Pessimism* | 0.98 (0.09) | 1.38 (0.10) | -0.22 |  |
| *Item 12 No future* | 1.22 (0.12) | 1.44 (0.15) | -0.15 |  |
| *Item 14 Not worth living* | 1.55 (0.18) | 1.29 (0.14) | -0.06 |  |
| *Item 19 Frequency cry* | 0.60 (0.07) | 0.52 (0.06) | 0.07 |  |
| *Item 38 Guilty* | 0.60 (0.06) | 1.01 (0.09) | -0.36 |  |
| *Item 39 Failure* | 1.29 (0.11) | 1.38 (0.12) | -0.05 |  |
| *Item 40 Feeling tense* | 0.74 (0.08) | 1.13 (0.09) | -0.16 |  |
| **Factor 2 Positive Symptoms** |  |  |  | -2.42 |
| *Item 2 Double meaning* | 0.70 (0.07) | 0.81 (0.08) | -0.08 |  |
| *Item 5 Messages from TV* | 0.61 (0.08) | 0.61 (0.08) | 0.00 |  |
| *Item 6 False appearance* | 0.68 (0.07) | 0.65 (0.06) | 0.02 |  |
| *Item 7 Being persecuted* | 1.01 (0.14) | 0.84 (0.13) | 0.08 |  |
| *Item 10 Conspiracy* | 1.14 (0.14) | 1.21 (0.17) | -0.06 |  |
| *Item 11 Being important* | 0.37 (0.06) | 0.64 (0.09) | -0.28 |  |
| *Item 13 Being special* | 0.38 (0.01) | 0.55 (0.07) | -0.17 |  |
| *Item 15 Telepathy* | 0.38 (0.06) | 0.60 (0.06) | -0.16 |  |
| *Item 17 influenced by devices* | 0.68 (0.11) | 0.45 (0.11) | 0.12 |  |
| *Item 20 Voodoo* | 0.36 (0.06) | 0.66 (0.08) | -0.30 |  |
| *Item 22 Odd looks* | 0.48 (0.06) | 0.80 (0.09) | -0.23 |  |
| *Item 24 Thought withdrawal* | 1.32 (0.24) | 1.44 (0.26) | -0.56 |  |
| *Item 26 Thought insertion* | 0.91 (0.11) | 1.18 (0.16) | -0.28 |  |
| *Item 28 Thought broadcasting* | 0.96 (0.12) | 1.11 (0.18) | -0.07 |  |
| *Item 30 Thought echo* | 1.03 (0.14) | 1.00 (0.16) | 0.01 |  |
| *Item 31 External control* | 1.08 (0.16) | 1.47 (0.26) | -0.17 |  |
| *Item 33 Verbal hallucinations* | 1.03 (0.15) | 0.83 (0.18) | 0.10 |  |
| *Item 34 Voices conversing* | 1.78 (0.48) | 1.51 (0.81) | -0.13 |  |
| *Item 41 Capgras* | 1.25 (0.36) | 1.07 (0.31) | 0.08 |  |
| *Item 42 Visual hallucinations* | 0.81 (0.16) | 1.13 (0.19) | -0.34 |  |
| **Factor 3 Negative Symptoms** |  |  |  | -0.05 |
| *Item 3 Lack of enthusiasm* | 0.99 (0.08) | 1.02 (0.09) | 0.02 |  |
| *Item 4 Not talkative* | 0.67 (0.06) | 0.58 (0.06) | 0.08 |  |
| *Item 8 No emotion* | 0.66 (0.07) | 0.54 (0.06) | 0.11 |  |
| *Item 16 No interest in others* | 0.69 (0.06) | 0.73 (0.07) | -0.03 |  |
| *Item 18 lack of motivation* | 1.07 (0.09) | 1.05 (0.08) | 0.01 |  |
| *Item 21 No energy* | 0.96 (0.08) | 0.91 (0.08) | -0.02 |  |
| *Item 23 Empty mind* | 0.52 (0.07) | 0.56 (0.07) | -0.02 |  |
| *Item 25 Lack of activity* | 1.04 (0.08) | 1.06 (0.09) | -0.01 |  |
| *Item 27 Blunted feelings* | 1.02 (0.09) | 0.91 (0.08) | -0.09 |  |
| *Item 29 Lack of spontaneity* | 0.86 (0.07) | 0.78 (0.06) | -0.07 |  |
| *Item 32 Blunted emotions* | 0.95 (0.08) | 1.03 (0.09) | -0.06 |  |
| *Item 35 Lack of hygiene* | 0.73 (0.08) | 0.70 (0.09) | 0.01 |  |
| *Item 36 Unable to terminate* | 0.89 (0.07) | 0.71 (0.07) | 0.11 |  |
| *Item 37 Lack of hobby* | 0.79 (0.08) | 0.78 (0.08) | 0.01 |  |
