# Supplementary material for: Does Assessment Type Matter? A Measurement Invariance Analysis of Online and Paper and Pencil Assessment of the Community Assessment of Psychic Experiences (CAPE)
Source: PLoS One. 2014 Jan 22;9(1):e84011. doi: 10.1371/journal.pone.0084011 (PMC3898946; doi:10.1371/journal.pone.0084011)
Supplement: Table S3 — Free parameters and Fit indices CFA analyses Internet and Paper sample matched for age. (DOC) [file pone.0084011.s003.doc]

**Table S3.** Free parameters and Fit indices CFA analyses Internet and Paper sample matched for age.

| **CFA Analysis** | **Number of Free Parameters** | **χ² (df)** | **RMSEA** | **CFI** | **TLI** |
| --- | --- | --- | --- | --- | --- |
| **Paper n = 793** | **119 free parameters;** | **477.57 (166)**** | **0.06** | **0.87** | **0.95** |
|  | 42 factor loadings |  |  |  |  |
|  | 72 thresholds |  |  |  |  |
|  | 3 factor covariances |  |  |  |  |
|  | 2 residual correlations |  |  |  |  |
| **Internet n = 793** | **119 free parameters;** | **520.63 (175)**** | **0.06** | **0.88** | **0.95** |
|  | 42 factor loadings |  |  |  |  |
|  | 72 thresholds |  |  |  |  |
|  | 3 factor covariances |  |  |  |  |
|  | 2 residual correlations |  |  |  |  |

*Note: CFA = Confirmatory Factor Analysis. df = degrees of freedom.* ******** *p < 0.001*
