# Supplementary material for: Does Assessment Type Matter? A Measurement Invariance Analysis of Online and Paper and Pencil Assessment of the Community Assessment of Psychic Experiences (CAPE)
Source: PLoS One. 2014 Jan 22;9(1):e84011. doi: 10.1371/journal.pone.0084011 (PMC3898946; doi:10.1371/journal.pone.0084011)
Supplement: Table S5 — Factor loadings, SIDS and STDS of the least restricted 3 factor model of the measurement invariance analysis for categorical data of the Paper and Internet sample matched for gender. Total STDS = −5.47. (DOC) [file pone.0084011.s005.doc]

**Table S5. Factor loadings, SIDS and STDS of the least restricted 3 factor model of the measurement invariance analysis for categorical data of the Paper and Internet sample matched for gender. Total STDS =-5.47.**

| **CAPE** | **Internet sample** | **Paper sample** | **SIDS** | **STDS** |
| --- | --- | --- | --- | --- |
| **Factor 1 (Depression)** | **Factor loadings (s.e.)** | **Factor loadings (s.e.)** |  | **-1.16** |
| *Item 1 Sad* | **1.05 (0.09)** | **1.41 (0.10)** | **-0.18** |  |
| *Item 9 Pessimism* | **0.83 (0.06)** | **1.30 (0.08)** | **-0.31** |  |
| *Item 12 No future* | **1.34 (0.11)** | **1.36 (0.13)** | **-0.01** |  |
| *Item 14 Not worth living* | **1.34 (0.15)** | **1.32 (0.13)** | **-0.00** |  |
| *Item 19 Frequency cry* | **0.37 (0.05)** | **0.50 (0.05)** | **-0.12** |  |
| *Item 38 Guilty* | **0.71 (0.07)** | **1.09 (0.08)** | **-0.33** |  |
| *Item 39 Failure* | **1.40 (0.12)** | **1.43 (0.11)** | **-0.01** |  |
| *Item 40 Feeling tense* | **0.71 (0.06)** | **1.16 (0.08)** | **-0.20** |  |
| **Factor 2 Positive Symptoms** |  |  |  | **-2.97** |
| *Item 2 Double meaning* | **0.63 (0.06)** | **0.81 (0.07)** | **-0.14** |  |
| *Item 5 Messages from TV* | **0.56 (0.07)** | **0.66 (0.07)** | **-0.04** |  |
| *Item 6 False appearance* | **0.73 (0.07)** | **0.63 (0.06)** | **0.06** |  |
| *Item 7 Being persecuted* | **0.83 (0.10)** | **0.89 (0.13)** | **-0.02** |  |
| *Item 10 Conspiracy* | **0.91 (0.09)** | **1.26 (0.16)** | **-0.32** |  |
| *Item 11 Being important* | **0.39 (0.05)** | **0.64 (0.08)** | **-0.25** |  |
| *Item 13 Being special* | **0.44 (0.05)** | **0.53 (0.06)** | **-0.09** |  |
| *Item 15 Telepathy* | **0.52 (0.05)** | **0.56 (0.05)** | **-0.04** |  |
| *Item 17 influenced by devices* | **0.62 (0.08)** | **0.50 (0.10)** | **0.06** |  |
| *Item 20 Voodoo* | **0.52 (0.06)** | **0.57 (0.07)** | **-0.05** |  |
| *Item 22 Odd looks* | **0.61 (0.06)** | **0.81 (0.08)** | **-0.12** |  |
| *Item 24 Thought withdrawal* | **0.87 (0.11)** | **1.43 (0.22)** | **-0.56** |  |
| *Item 26 Thought insertion* | **1.06 (0.12)** | **1.22 (0.14)** | **-0.15** |  |
| *Item 28 Thought broadcasting* | **0.50 (0.07)** | **1.07 (0.14)** | **-0.26** |  |
| *Item 30 Thought echo* | **0.65 (0.08)** | **0.93 (0.12)** | **-0.12** |  |
| *Item 31 External control* | **0.83 (0.11)** | **1.36 (0.19)** | **-0.23** |  |
| *Item 33 Verbal hallucinations* | **1.00 (0.12)** | **1.02 (0.18)** | **-0.01** |  |
| *Item 34 Voices conversing* | **1.50 (0.28)** | **2.04 (1.08)** | **-0.24** |  |
| *Item 41 Capgras* | **0.95 (0.19)** | **1.17 (0.28)** | **-0.10** |  |
| *Item 42 Visual hallucinations* | **0.83 (0.10)** | **1.20 (0.18)** | **-0.35** |  |
| **Factor 3 Negative Symptoms** |  |  |  | **-1.34** |
| *Item 3 Lack of enthusiasm* | **1.00 (0.08)** | **0.95 (0.07)** | **-0.10** |  |
| *Item 4 Not talkative* | **0.55 (0.05)** | **0.57 (0.05)** | **-0.03** |  |
| *Item 8 No emotion* | **0.65 (0.05)** | **0.48 (0.05)** | **-0.03** |  |
| *Item 16 No interest in others* | **0.63 (0.05)** | **0.68 (0.06)** | **0.01** |  |
| *Item 18 lack of motivation* | **0.80 (0.06)** | **1.05 (0.07)** | **-0.11** |  |
| *Item 21 No energy* | **0.77 (0.05)** | **0.91 (0.07)** | **-0.13** |  |
| *Item 23 Empty mind* | **0.51 (0.06)** | **0.58 (0.06)** | **-0.12** |  |
| *Item 25 Lack of activity* | **0.78 (0.06)** | **1.05 (0.08)** | **-0.10** |  |
| *Item 27 Blunted feelings* | **1.02 (0.08)** | **0.85 (0.07)** | **-0.17** |  |
| *Item 29 Lack of spontaneity* | **0.77 (0.06)** | **0.73 (0.05)** | **-0.08** |  |
| *Item 32 Blunted emotions* | **1.05 (0.08)** | **1.02 (0.08)** | **-0.26** |  |
| *Item 35 Lack of hygiene* | **0.66 (0.07)** | **0.70 (0.08)** | **-0.04** |  |
| *Item 36 Unable to terminate* | **0.69 (0.06)** | **0.74 (0.06)** | **-0.08** |  |
| *Item 37 Lack of hobby* | **0.72 (0.06)** | **0.77 (0.07)** | **-0.10** |  |
