# Supplementary material for: Does Assessment Type Matter? A Measurement Invariance Analysis of Online and Paper and Pencil Assessment of the Community Assessment of Psychic Experiences (CAPE)
Source: PLoS One. 2014 Jan 22;9(1):e84011. doi: 10.1371/journal.pone.0084011 (PMC3898946; doi:10.1371/journal.pone.0084011)
Supplement: Table S7 — Free parameters and Fit indices MI analyses Internet and Paper sample matched for gender. (DOC) [file pone.0084011.s007.doc]

**Table S7:** Free parameters and Fit indices MI analyses Internet and Paper sample matched for gender.

| **MI Analysis** | **Nr. of Free Parameters** | **χ² (df)** | **RMSEA** | **CFI** | **TLI** | **χ² difftest**^a^ |
| --- | --- | --- | --- | --- | --- | --- |
| **Configural model** | **238 free parameters;** | **1336.83 (416)**** | **0.05** | **0.87** | **0.95** |  |
| ***Group 1 paper*** | 42 factor loadings |  |  |  |  |  |
|  | 72 thresholds |  |  |  |  |  |
|  | 2 residual correlations |  |  |  |  |  |
|  | 3 factor covariances |  |  |  |  |  |
| ***Group 2 Internet*** | 42 factor loadings |  |  |  |  |  |
|  | 72 thresholds |  |  |  |  |  |
|  | 2 residual correlations |  |  |  |  |  |
|  | 3 factor covariances |  |  |  |  |  |
| **Metric invariance** | **197 free parameters;** | **1335.12 (405)**** | **0.05** | **0.87** | **0.95** | **124.03 (27)**** |
| ***Group 1 paper*** | 42 factor loadings |  |  |  |  |  |
|  | 72 thresholds |  |  |  |  |  |
|  | 2 residual correlations |  |  |  |  |  |
|  | 3 factor covariances |  |  |  |  |  |
| ***Group 2 Internet*** | 27 thresholds |  |  |  |  |  |
|  | 6 factor (co)variances |  |  |  |  |  |
|  | 3 factor means |  |  |  |  |  |
|  | 42 residual variances |  |  |  |  |  |
| **Strong invariance** | **170 free parameters;** | **1578.54 (409)**** | **0.06** | **0.84** | **0.93** | **657.84 (23)**** |
| ***Group 1 paper*** | 42 factor loadings |  |  |  |  |  |
|  | 72 thresholds |  |  |  |  |  |
|  | 2 residual correlations |  |  |  |  |  |
|  | 3 factor covariances |  |  |  |  |  |
| ***Group 2 Internet*** | 6 factor (co)variances |  |  |  |  |  |
|  | 3 factor means |  |  |  |  |  |
|  | 42 residual variances |  |  |  |  |  |
| **Strict invariance** | **128 free parameters;** | **1383.99 (365)**** | **0.06** | **0.86** | **0.93** | **108.44 (34)**** |
| ***Group 1 paper*** | 42 factor loadings |  |  |  |  |  |
|  | 72 thresholds |  |  |  |  |  |
|  | 2 residual correlations |  |  |  |  |  |
|  | 3 factor covariances |  |  |  |  |  |
| ***Group 2 Internet*** | 6 factor (co)variances |  |  |  |  |  |
|  | 3 factor means |  |  |  |  |  |

*Note: MI = Measurement Invariance. df = degrees of freedom. Metric invariance; model fit compared to fit configural model. Strong invariance; model fit compared to fit Metric invariance model. Strict invariance; model fit compared to fit Strong invariance model.*

*^a^ χ² difftest* *was conducted in Mplus by use of WLSMV estimator. ** p < 0.001.*
